# A 3D Printed Anatomically Pre-Contoured Plate for the Treatment of Y-T Humeral Condylar Fractures: A Feline Cadaveric Study

**DOI:** 10.3390/ani14040537

**Published:** 2024-02-06

**Authors:** Piotr Trębacz, Jan Frymus, Anna Barteczko, Mateusz Pawlik, Aleksandra Kurkowska, Michał Czopowicz

**Affiliations:** 1 Department of Surgery and Anaesthesiology of Small Animals, Institute of Veterinary Medicine, Warsaw University of Life Sciences-SGGW, Nowoursynowska 159 C Street, 02-776 Warsaw, Poland; 2 CABIOMEDE Ltd., Karola Olszewskiego 21 Street, 25-663 Kielce, Poland; anna.barteczko@cabiomede.com (A.B.); mateusz.pawlik@cabiomede.com (M.P.); aleksandra.kurkowska@cabiomede.com (A.K.); 3 Department of Biomaterials and Medical Devices Engineering, Faculty of Biomedical Engineering, Silesian University of Technology, Roosevelta 40 Street, 41-800 Zabrze, Poland; 4 Division of Veterinary Epidemiology and Economics, Institute of Veterinary Medicine, Warsaw University of Life Sciences-SGGW, Nowoursynowska 159 C Street, 02-776 Warsaw, Poland; michal_czopowicz@sggw.edu.pl

**Keywords:** cat, bicondylar humeral fracture, bone plate, additive manufacturing

## Abstract

**Simple Summary:**

Y-T condylar fractures are intra-articular fractures of the distal humerus characterised by a central intercondylar split and proximal extension of the fracture line through both the medial and lateral epicondyles. Such fractures are rare in the feline elbow and are perhaps the most difficult to treat. Because of the non-perforated supratrochlear foramen, feline bicondylar fractures are often associated with high-energy trauma and usually involve comminution of the epicondylar region. The humeral condyle needs to be anatomically reduced and stabilised with a screw. Plates are then most commonly added to address the supracondylar portion of the fracture. Locking internal fixation, which eliminates friction between plate and bone, may offer advantages in the repair of humeral condylar fractures. Human anatomically pre-contoured locking compression plate systems have been used to repair distal humeral fractures in humans with good to excellent clinical results. The aim of this study was to describe the development of a 3D printed anatomically pre-contoured locking plate and the use of such a plate for the reduction and stabilisation of bicondylar humeral fractures in isolated feline forelimbs. The results obtained suggest that such a plate may have application in the stabilisation of complex Y-T humeral fractures in cats.

**Abstract:**

(1) Background: Anatomically pre-contoured plates usually require only minimal or even no intraoperative contouring. For complex cases, such plates also assist the surgeon as an anatomical template during fracture reduction. In this study, we present our experience of using a 3D printing technology for the treatment of bicondylar humeral fractures in feline cadavers. (2) Methods: Surgeries were performed on 15 pairs of front limbs amputated at the scapula. The limbs were obtained from 15 adult cats without obvious pathology of the skeleton. After flexion of the elbow and subperiosteal elevation of the anconeus muscle, the humeral Y-T fractures were created using a bone chisel and mallet. A custom-made anatomically pre-contoured interlocking plate was used to reduce and stabilise the medial aspect of the humeral condyle to the humeral diaphysis. After reduction of the humeral condyle, a positional locking screw was then inserted from the medial to the lateral side and a straight 2.4/2.7 interlocking bone plate was used to stabilise the lateral part of the condyle to the humeral diaphysis. (3) Results: The length of the humerus ranged from 98.2 to 107.0 mm and did not differ significantly between the left and right bone. The diameter of the isthmus of the humeral condyle ranged from 5.2 to 5.5 mm and did not differ significantly between the left and right bone. In all 30 limbs, bicondylar fracture was accompanied by epicondylar comminution. In 7/30 limbs (4 left, 3 right) the fracture of the humeral shaft was also present. In the left limbs, the postoperative articular surface defect of the humeral condyle was small (<1 mm) in 11/15 cases, moderate (1–2 mm) in 2/15 cases and large (>2 mm) in 2/15 cases in which the condylar screw was incorrectly inserted. In the right limbs, the postoperative articular surface defect of the humeral condyle was small (<1 mm) in 14/15 cases and moderate (1–2 mm) in 1 case. (4) Conclusions: 3D printing and the technology of metal powder sintering offers a wide range of possibilities for the development of new surgical implants. The anatomically pre-contoured bone plate appears to be a valuable tool in the reduction and stabilisation of Y-T humeral fractures in adult domestic cats weighing 3.0 to 4.5 kg.

## 1. Introduction

The feline humeral condyle is relatively uniform and lacks the supratrochlear foramen. Fractures of the humeral condyle are therefore rare in cats [1,2,3]. Where such injuries have been reported, they are often associated with high-energy trauma, so condylar fractures are usually accompanied by supracondylar comminution [2,4]. These fractures are also called bicondylar, dicondylar, intercondylar or Y-T fractures. In general, surgical management involves a combination of medial and lateral approaches to the condyle and stable osteosynthesis. As part of the fixation, a transcondylar screw is recommended. Intercondylar humeral fractures require accurate anatomical reduction to restore joint congruity [5]. Reconstruction of a comminuted supracondylar fracture may not be possible and, at best, involves intricate surgical manipulation, which can have a negative impact on fracture biology and subsequent bone healing. Management of the supracondylar component by internal fixation has been described in a series of cats. Macias et al. [2] describe the use of a transcondylar lag screw and paired supracondylar neutralisation plates to treat this fracture configuration in five cats. Major complications were reported in two of the four cats which had non-reconstructable fractures. These included early implant failure and loss of elbow range of motion, resulting in significant lameness and debilitation. Early implant failure was thought to be a result of the limited number of cortices that could be engaged in the distal fracture segment. A more biological approach has also been described, through the use of linear circular external skeletal fixator systems to treat juxta-articular fracture configurations [6]. The outcome of one case was excellent and the outcome in the second case was fair. Better results were attained by Silva et al. [7], who treated four cats with Y-T humeral fractures with a hybrid external circular fixator.

Bone plates are the most commonly used internal fixation devices, and they are manufactured in various sizes and shapes to accommodate different patients [8]. However, a challenge arises when there is a geometrical mismatch between the size and shape of the patient’s bone and the standard plate implant [8,9,10]. In such cases, accurately positioning the plate becomes difficult, and the treatment may be complicated by inadequate load transfer during the bone healing process. Anatomically pre-contoured plates are now the standard of care for the internal fixation of periarticular fractures in humans. Often, they require minimal or no intraoperative contouring. For complex cases, such plates also assist the surgeon as an anatomical template during fracture reduction [10,11,12,13]. To address this issue, personalised plate implants have been introduced. Pre-contoured interlocking compression plate systems have been used in the repair of distal humeral fractures in humans with good to excellent clinical outcomes [14,15]. These implants are specifically customised to align with the patient’s bone anatomy and meet the requirements of the surgeon [8,9,10,11,12].

Rapid prototyping, also known as three-dimensional (3D) printing, has become a helpful procedure in many medical and veterinary fields, and its advantages in fracture treatment have been highlighted [8,9,16]. 3D printing technology enables the rapid construction of accurate, complete, custom implants and aiming devices.

In this study, we present our experience of using 3D printing technology for the treatment of bicondylar humeral fractures in feline cadavers. We created a medially inserted anatomically pre-contoured interlocking bone plate and hypothesised that such a plate would be a valuable tool for the reduction and stabilisation of humeral Y-T fractures.

## 2. Materials and Methods

### 2.1. Creation of 3D Printed Bone Plate

The process began with a detailed analysis and segmentation of thirty computed tomography (CT) scans of European domestic cats with no apparent forelimb pathology. The animals had been scanned for reasons unrelated to this study (whole body scans following road traffic accidents). The humeri and elbows were reconstructed using a bone algorithm for evaluation and measurement. Geomagic Freeform software (https://www.3dsystems.com/ accessed on 1 December 2023, Circle Rock Hill, SC, Canada) was used to create an averaged 3D model of the left and right humerus, which served as the basis for further design stages.

The initial design stage of the plate involved the creation of a 2D drawing and a 3D model showing the geometric outline of the area to be stabilised by the implant. Then, an analysis of the plate geometry and cross-sections was performed. A thickness map of the plate section was generated with specified thresholds (minimum 2 mm), allowing corrections to be made by thickening areas where the thickness was below the minimum. At this stage, the positions of the holes for the screws and Kirschner wires were determined in the target geometry of the plate, and undercuts were generated to limit contact with the bone surface in the part encompassing the shaft.

**Figure 1:**
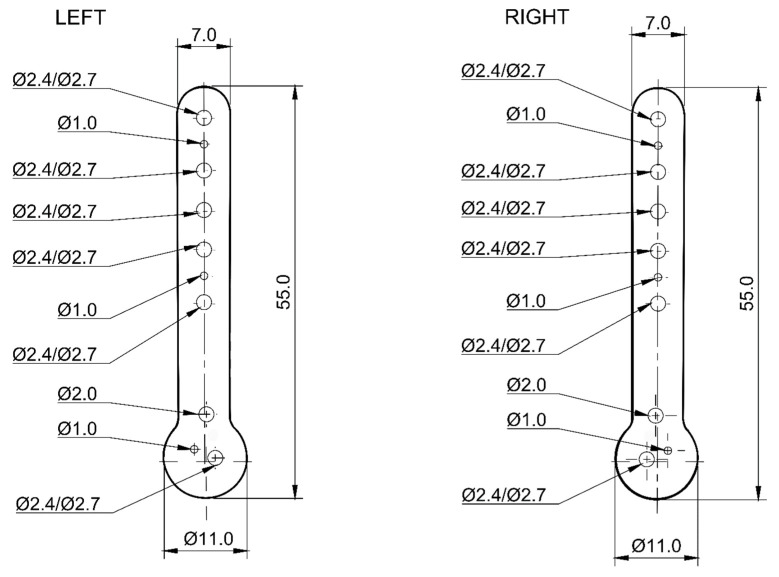
Diagram of anatomically pre-contoured interlocking bone plates showing the distribution of holes for locking screws and Kirschner wires (dimensions in mm).

A spatial stereolitography (STL) grid for 3D printing was generated and the model was prepared for printing. The slices of the model were generated in the Chitubox slicer software (https://cc.chitubox.com accessed on 1 December 2023, Shenzhen, China), which is designed for 3D printing. In the prototyping phase, high-resolution light-curing resin prototypes (XY resolution = 22 µm, Z-axis layer thickness 50 µm) were made to test the geometry of the new plate shape. The pre-prototypes were tested on a 3D printed bone models (left and right) of a feline humerus and right forelimb amputee from an adult male domestic cat weighing 4.0 kg following a traumatic paw amputation. Adapting the model to the bone allowed the determination of the required plate thickness and the final positions of the holes for the bone screws and Kirschner wires as follows:

- in the shaft: 5 for Ø2.4/2.7 mm locking screws and 2 for Ø1.0 mm Kirschner wires;
- in the head: 1 for Ø2.0 mm locking screw, 1 for Ø2.4/2.7 mm locking screw and 1 for Ø1.0 mm Kirschner wire (Figure 1).

Following positive verification of the plate’s utility, final prototypes were fabricated in metal using Ti6Al4V titanium alloy powder in accordance with the ISO medical standard [17] in a process certified according to ISO [18] using direct laser metal sintering (DLMS) technology.

**Figure 2:**
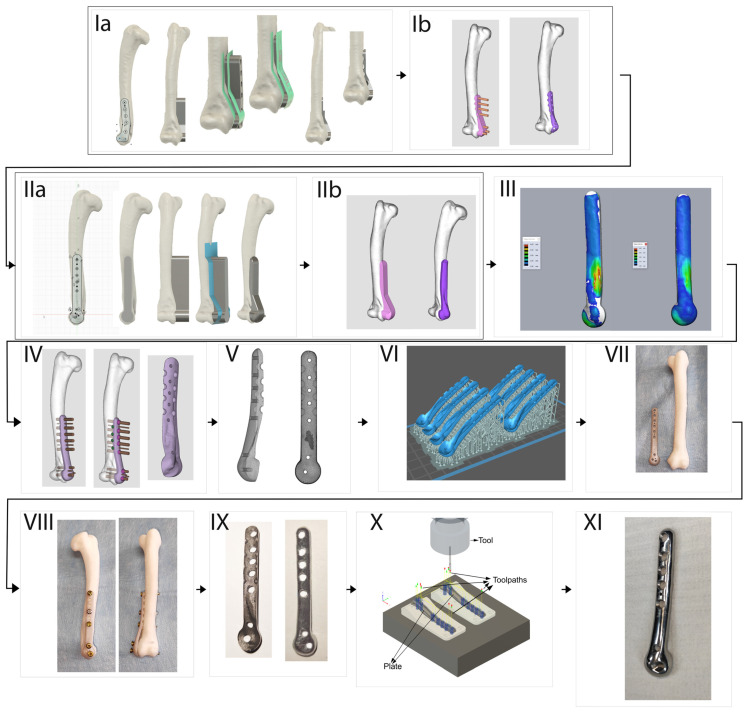
Workflow showing the creation of a 3D printed anatomically pre-contoured interlocking bone plate for the reduction and stabilisation of feline Y3D-T humerus fractures. (**Ia**) Preliminary design—plate outline drawing and solid model. (**Ib**) Fitting the model to the bone, determination of the holes’ position. (**IIa**) Drawing of the plate outline and solid model with applied corrections after consultation with the surgeon. (**IIb**) Fitting updated plate model to the shape of the bone. (**III**) Analysis of the cross-sectional geometry of the plate, offsetting areas with thickness less than 2mm (white regions). (**IV**) Determination of holes’ position in the plate and applying undercuts limiting contact with the bone surface. (**V**) Generation of STL spatial mesh. (**VI**) Preparation of machine code for production of polymer prototypes in 3D printing software (Chitubox slicer, https://cc.chitubox.com accessed on 1 December 2023). (**VII**) Printed from photopolymer resin prototype of the implant for first functional tests. (**VIII**) Testing of the polymer plate on synthetic bone. (**IX**) Raw print from Ti6Al4V alloy using direct laser metal sintering technology. (**X**) Preparation of CAM CNC machine code, milling of Kirchner wire holes and locking screw sockets. (**XI**) Final product—plate after CNC machining and polishing of the outer surface.

The final stage of the project involved the post-processing of the plate. This included mechanical removal of the supports, manual pre-grinding and computerised numerical control (CNC) milling of the holes for the locking screw sockets. The work was completed by removing the sharp edges created by the CNC machining and final polishing of the plate face (Figure 2).

### 2.2. Preparation of Surgical Specimens

Surgeries were performed on 15 pairs of front limbs amputated at the scapula. The limbs were obtained from 15 adult cats without obvious pathology of the skeleton—8 males and 7 females—aged from 2 to 17 years with the arithmetic mean (±SD) of 9.7 ± 3.5 years. Nine were domestic short haired (DSH) cats (60%), 5 domestic long haired (DLH) cats, and one Cornish Rex. Their body weight ranged from 3.0 to 4.5 kg with the arithmetic mean (±SD) of 3.7 ± 0.5 years. The animals died or were euthanised for reasons unrelated to this study. Ethical approval was not required for this study according to Polish legal regulations (The Act of the Polish Parliament of 15 January 2015 on the Protection of Animals Used for Scientific or Educational Purposes, Journal of Laws 2015, item 266). After flexion of the elbow and subperiosteal elevation of the anconeus muscle, the humeral Y-T fractures were created using a bone chisel and mallet.

### 2.3. Radiographic Assessment

Standard calibrated orthogonal radiographs of the amputated limbs were assessed before and after the Y-T fracture creation. For each limb, the length of the humerus, the diameter of the isthmus of the humeral condyle measured on the cranio–caudal radiographs and the fracture configuration were assessed. The implants and repair were assessed on postoperative radiographs. The accuracy of articular surface reduction and the resulting articular surface defect were measured and graded as small (<1 mm), moderate (1–2 mm) or large (>2 mm). In addition, alignment (axial and rotational alignment of the joints above and below the fracture), adjacency (adequate attachment of the main fracture fragments to the comminution zone) and apparatus (implants’ location, placement, security and size) were assessed.

### 2.4. Surgical Technique

**Figure 3:**
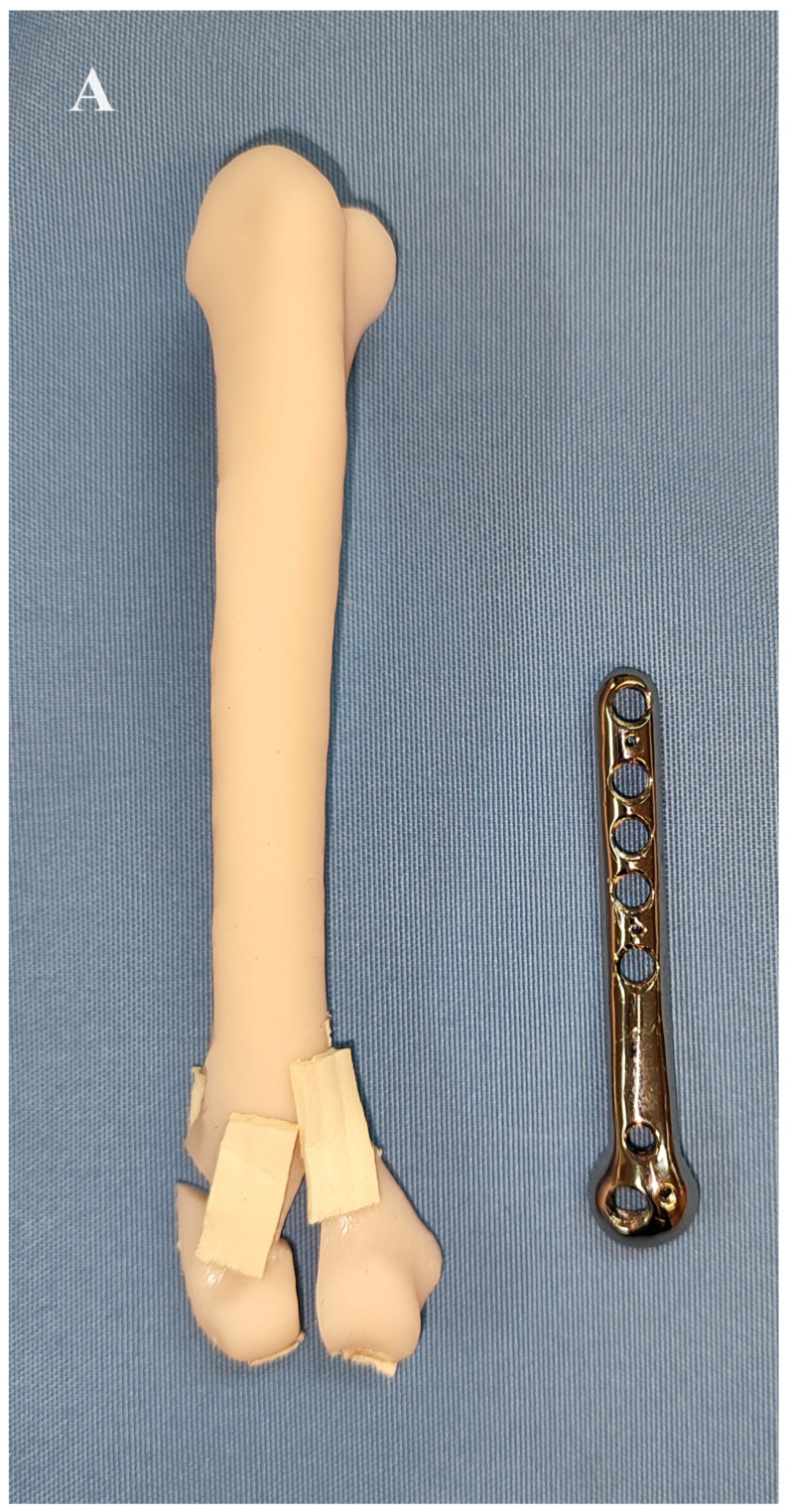

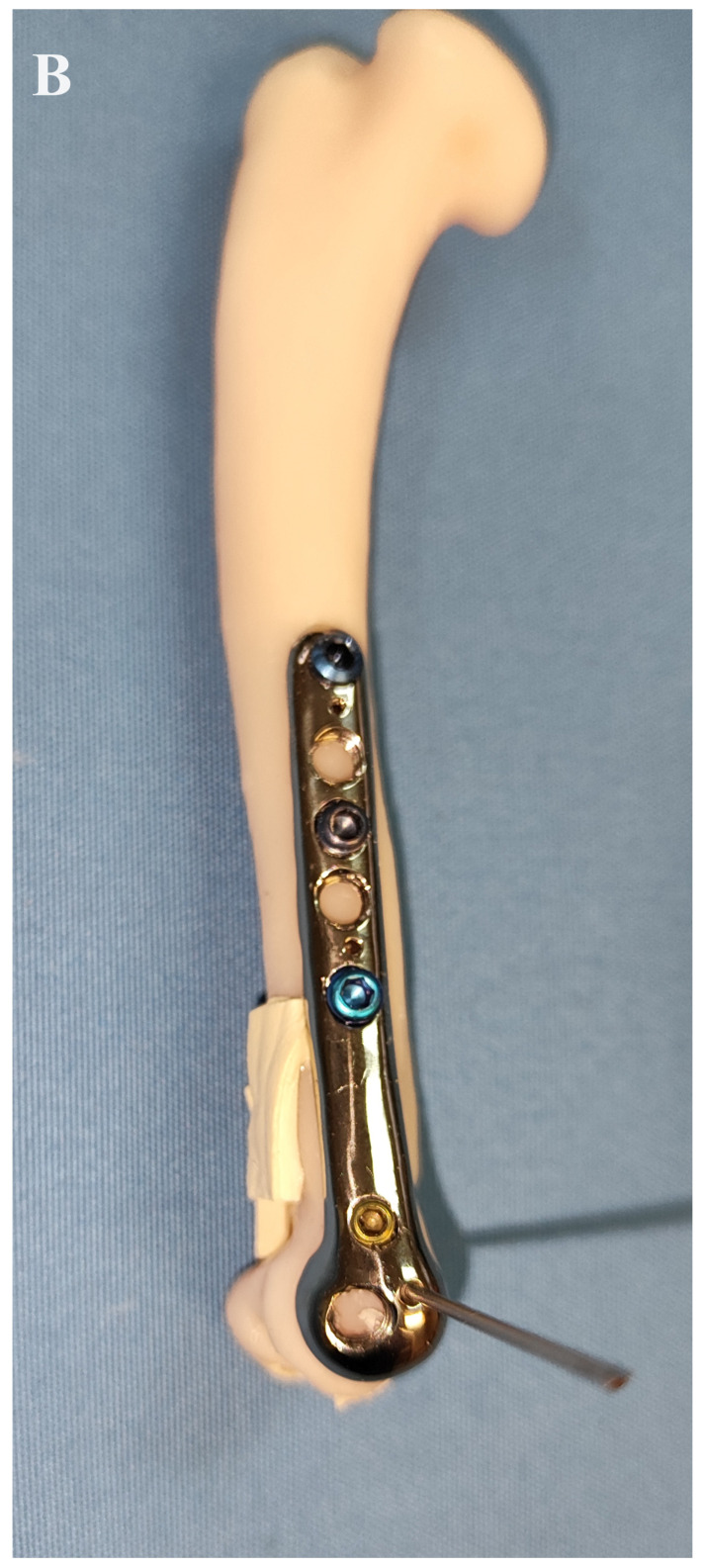

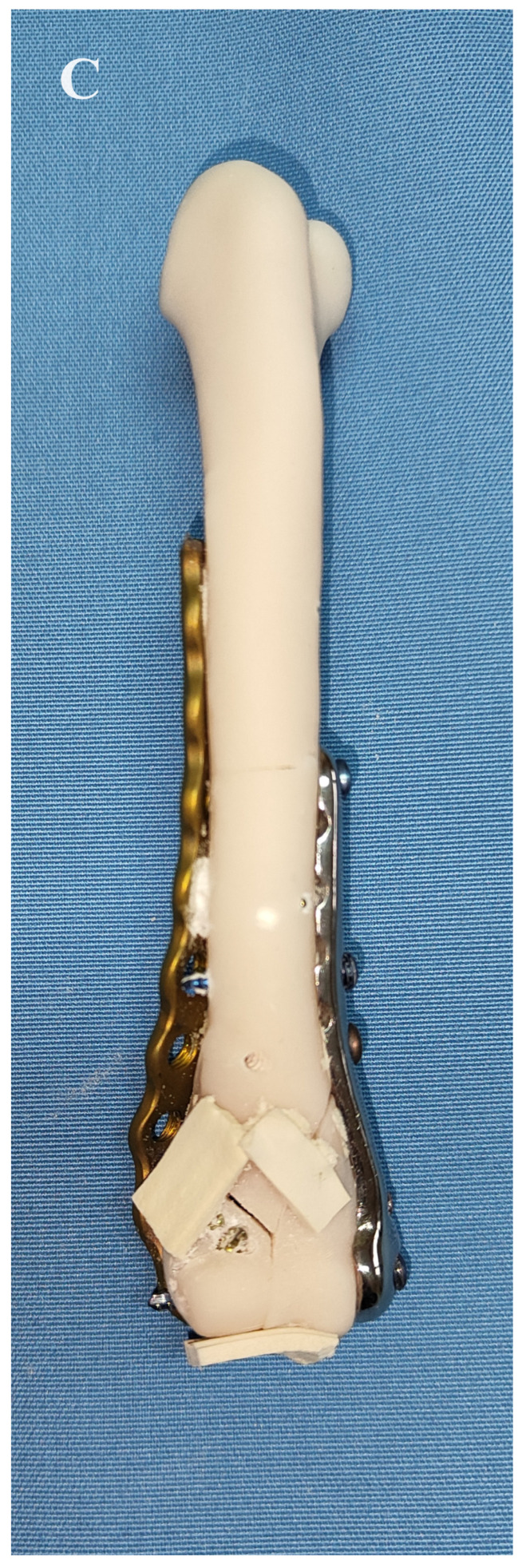
Reduction and stabilisation of the Y-T fracture of the right humerus on the 3D printed bone model. (**A**–**C**) After conversion of the bicondylar fracture to a monocondylar lateral fracture using the printed plate, the lateral part of the fracture was stabilised with a 2.4/2.7 locking plate.

**Figure 4:**
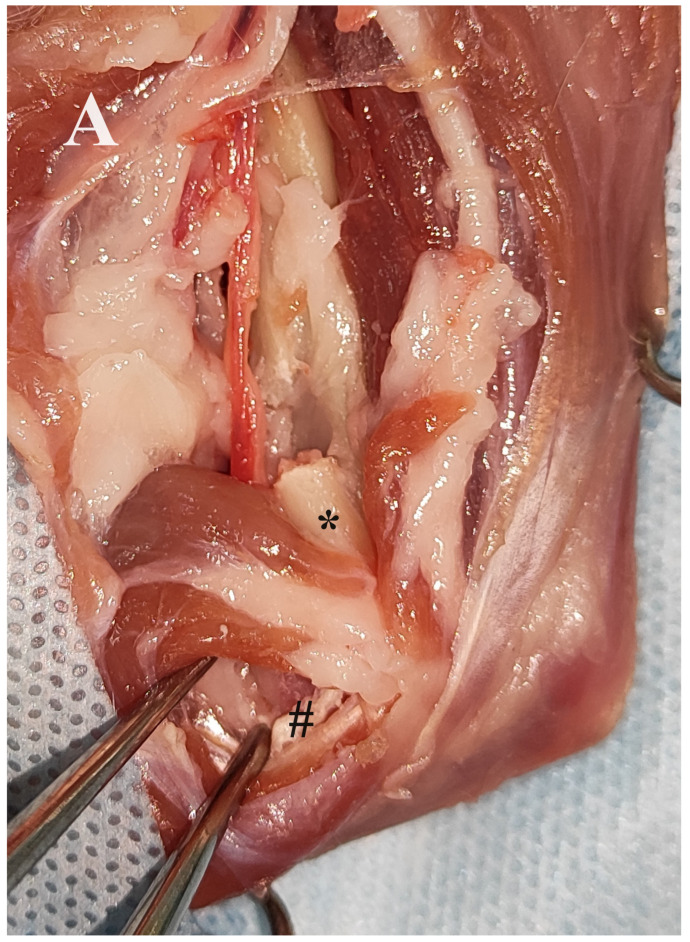

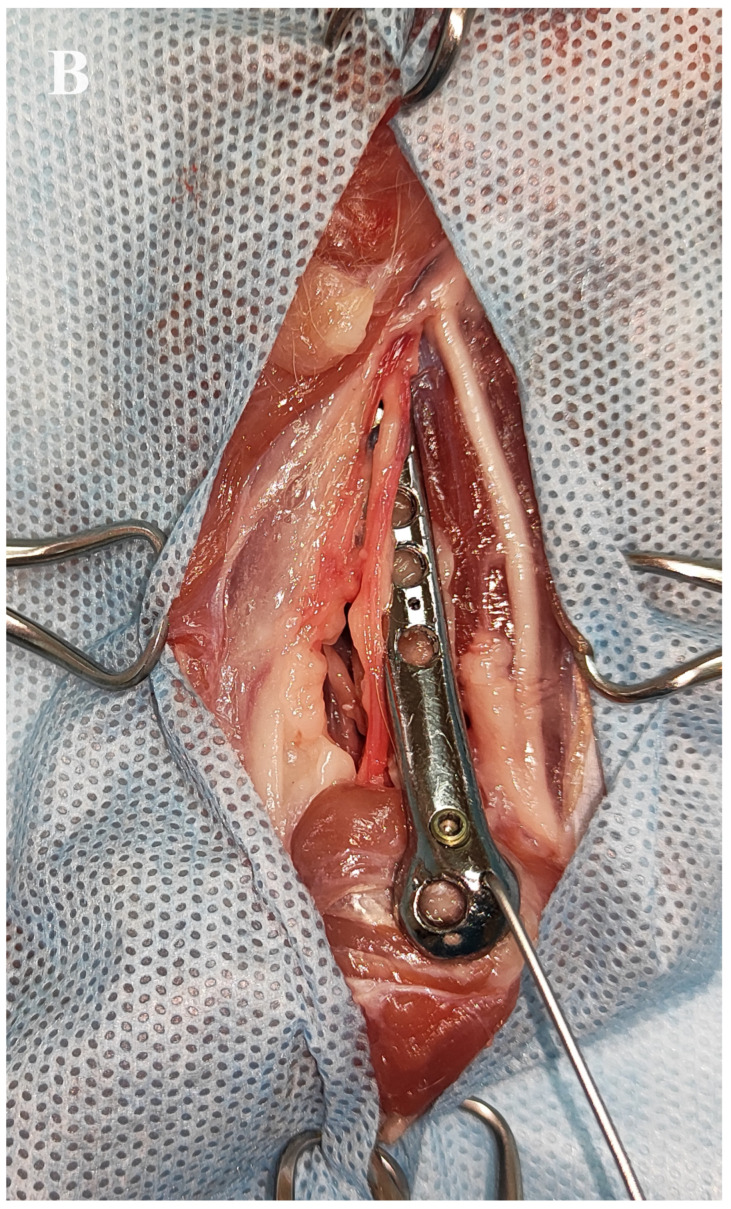

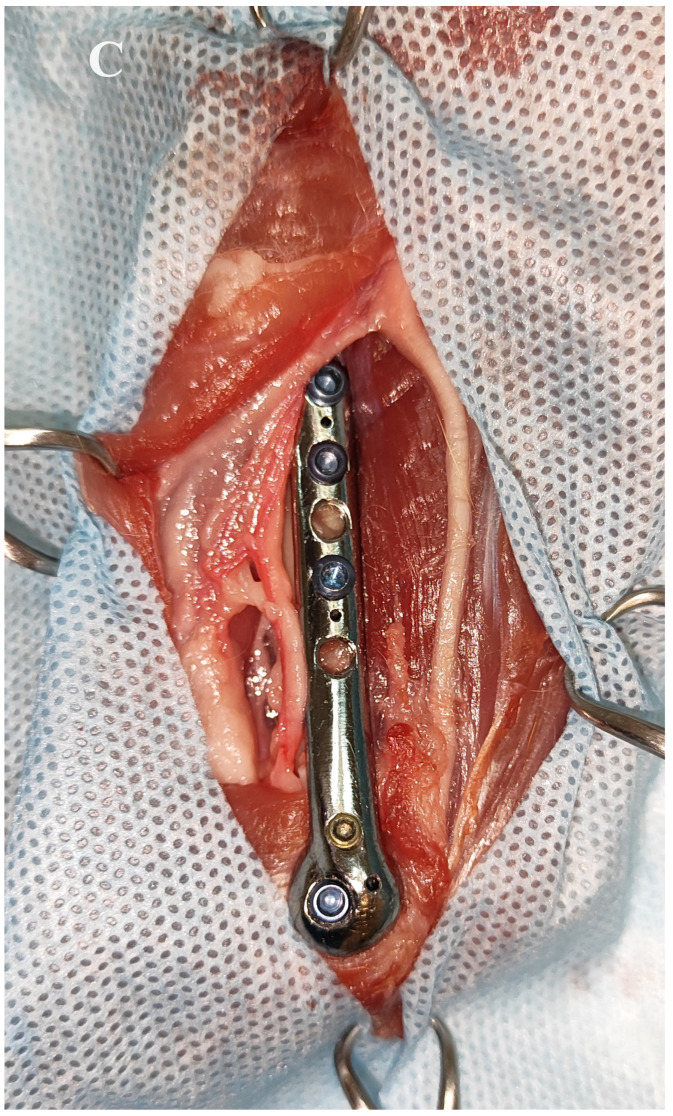

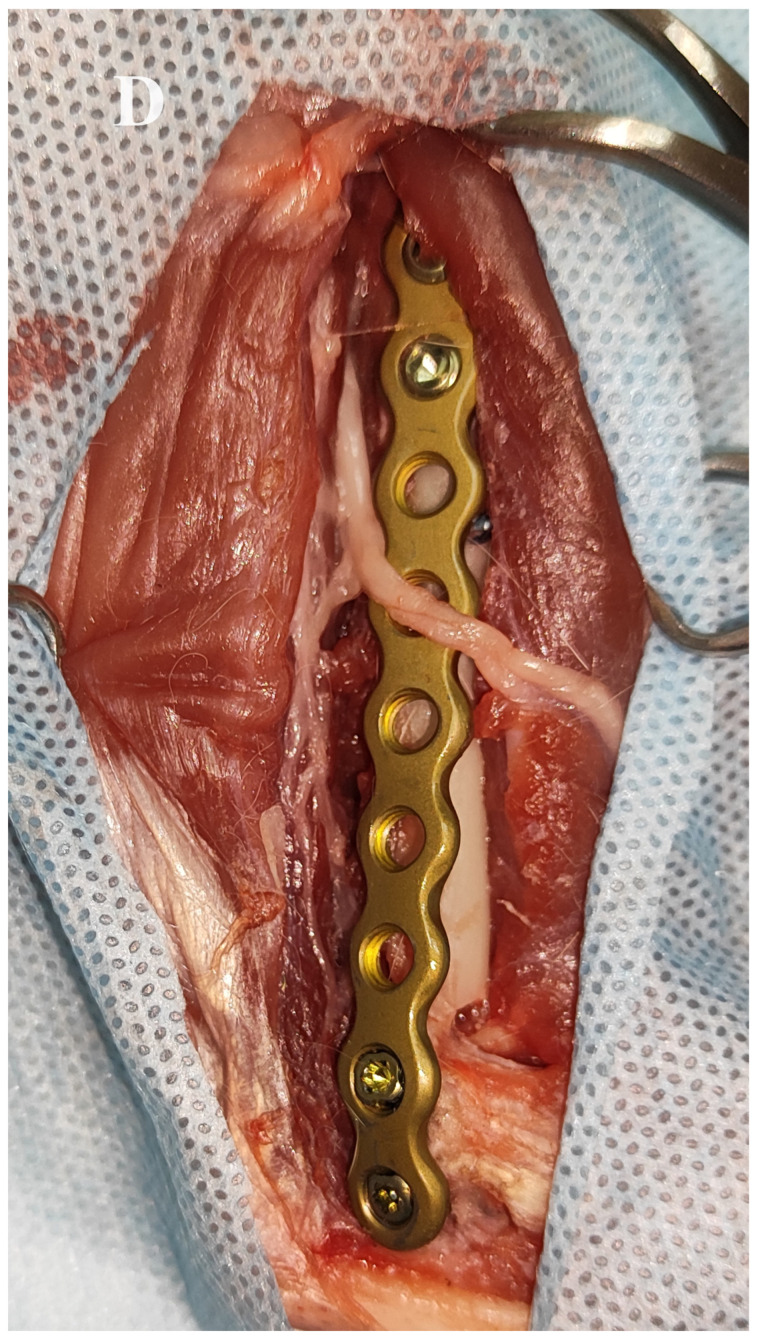
Medial approach to the right humerus and insertion of the 3D printed plate in an adult female domestic cat. (**A**,**B**) Exposure of the medial surface of the right humerus. Release of the brachial artery, brachial vein and median nerve from the supracondylar foramen after removal of its medial border (asterisk). Exposure of the medial part of the humeral condyle (grid) after dissection between the pronator teres muscle and the antebrachial flexor muscles. Conversion of the bicondylar fracture to a monocondylar fracture using the printed plate. (**C**,**D**) Final stabilisation of the medial and lateral part of the Y-T fracture.

**Figure 5:**
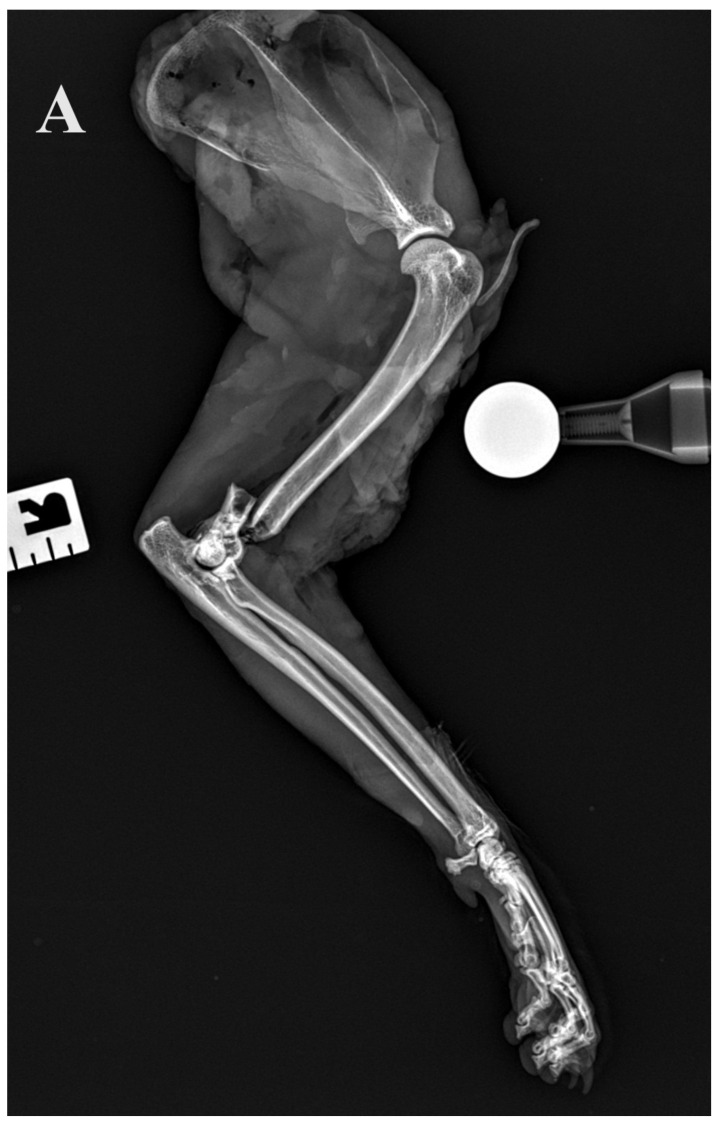

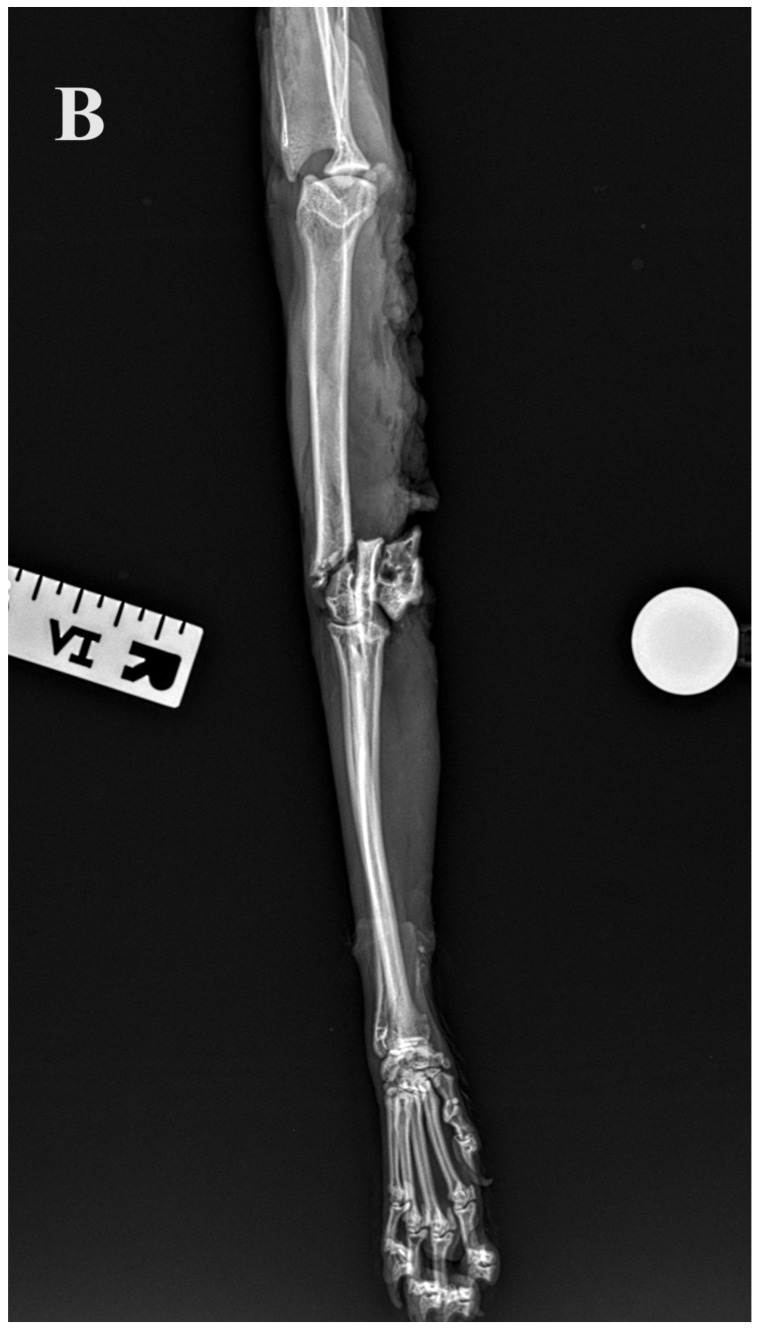

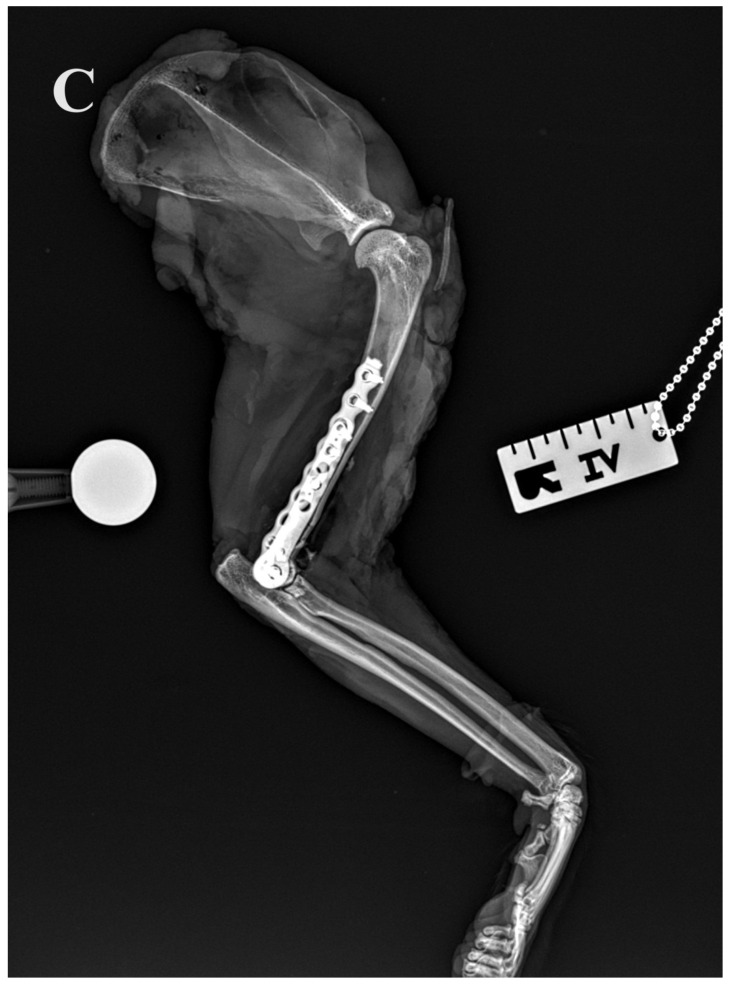

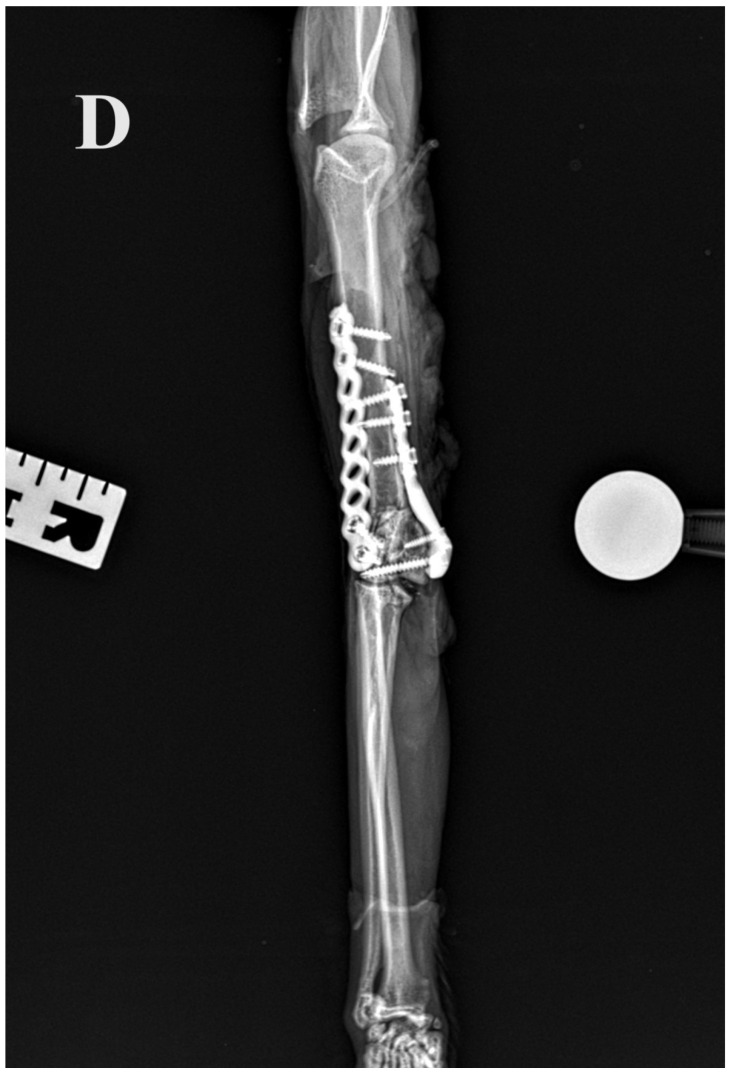
(**A**,**B**) Initial lateral and cranio–caudal radiographs of the right forelimb of a female domestic cat show a displaced Y-T fracture. (**C**,**D**) Postoperative lateral and cranio–caudal radiographs show a small articular surface defect (<1 mm) of humeral condyle and adequate alignment, adjacency and apparatus (2.0/2.4/2.7 3D printed plate, 2.4/2.7 straight interlocking bone plate and screws).

The fractures were exposed by combined medial and lateral approaches to the humerus. Care was taken to avoid injury to the neurovascular structures. In all procedures, a medial approach to the humerus was first performed to treat the medial condylar fracture in order to transform the bicondylar fracture into a lateral monocondylar fracture. To ensure good implant positioning and to avoid impingement of the neurovascular structures by the plate, cranial retraction of the brachial artery, vein and median nerve was performed after freeing these structures from the supracondylar foramen by removing its medial border with a rongeur as described by Maritato et al. [19]. In addition, the origin of the medial head of the triceps brachii muscle was transected and retracted caudally. The medial surface of the humeral condyle was partially exposed after combined blunt and sharp dissection between the pronator teres muscle and the antebrachial flexor muscles. Then, a custom-made anatomically pre-contoured interlocking plate was used to reduce and stabilise the medial aspect of the humeral condyle to the humeral diaphysis. The medial part of the humeral condyle was initially stabilised with a 1 mm Kirschner wire inserted through the medial epicondyle and a 2 mm locking screw (Iwet, Grabówka, Poland). The shaft of the plate was first stabilised with 1 mm Kirschner wires and then with 2.4 mm locking screws (Iwet, Grabówka, Poland) (Figure 3 and Figure 4). A lateral approach to the distal humerus allowed reduction and stabilisation of the intracondylar aspect of the fracture with pointed reduction forceps. A 2.7 mm preloaded positional locking screw was then inserted from the medial to the lateral side between pronator teres muscle and antebrachial flexors and a 2.4/2.7 interlocking bone plate (Medgal, Księżyno, Poland) was used to stabilise the lateral part of the condyle to the humeral diaphysis (Figure 5). In cases with additional humeral shaft fractures, cerclage wires were used to support the bone plates.

### 2.5. Statistical Methods

Numerical variables (age, body weight, humerus diameters) were examined for normality of distribution using the normal probability Q-Q plots and the Shapiro–Wilk W test. As the normality assumption was held, they were summarised using the arithmetic mean (M), standard deviation (±SD) and range. Variability of the humerus diameters was expressed as the coefficient of variation (CV) calculated as:

$$\mathrm{CV}=\frac{\mathrm{SD}}{M}\times 100\%$$

The 95% confidence interval (CI 95%) for the CV was calculated as follows [20]:

$$\mathrm{CI}\;95\%=\mathrm{CV}\pm 1.96\times \mathrm{CV}\times \sqrt{\frac{{\mathrm{CV}}^{2}+0.5}{n-1}}$$

where n denotes the number of analysed elements. Humerus diameters were compared between sides using the paired Student’s *t*-test. A significance level (α) was set at 0.05 and the analysis was performed in TIBCO Statistica 13.3 (TIBCO Software Inc., Palo Alto, CA, USA).

## 3. Results

After analysing CT scans of both humeri of thirty domestic cats, an average 3D model of the left and right humerus was created. The length was 104 mm and the diameter of the isthmus of the humeral condyle was 5.5 mm.

No radiographic abnormalities were found in the operated limbs. Length of the humerus ranged from 98.2 to 107.0 mm and did not differ significantly between the left (103.43 ± 2.64 mm) and right bone (103.47 ± 2.60 mm) (*p* = 0.373). The CV of the humerus length was 2.6% (CI 95%: 1.6%, 3.5%) for the left humerus and 2.5% (CI 95%: 1.6%, 3.5%) for the right humerus. The diameter of the isthmus of the humeral condyle ranged from 5.2 to 5.5 mm and did not differ significantly between the left (5.39 ± 0.10 mm) and right (5.39 ± 0.08 mm) bone (*p* = 0.999). The CV of the isthmus diameter was 1.8% (CI 95%: 1.2%, 2.5%) for the left humerus and 1.5% (CI 95%: 1.0%, 2.1%) for the right humerus.

In all 30 limbs, the bicondylar fracture was accompanied by epicondylar comminution. In 7/30 limbs (4 left, 3 right), a fracture of the humeral shaft was also present.

In the left limbs, the postoperative articular surface defect of the humeral condyle was small (<1 mm) in 11/15 cases, moderate (1–2 mm) in 2/15 cases and large (>2 mm) in 2/15 cases in which the condylar screw was inserted incorrectly. In the right limbs, the postoperative articular surface defect of the humeral condyle was small (<1 mm) in 14/15 cases and moderate (1–2 mm) in 1 case. In all cases, reduction and stabilisation of the fracture with the pre-contoured plate was found to be easy. With the exception of two left limbs where the condylar screw was inserted incorrectly, the general alignment of the humerus fracture, the general adjacency of the fracture fragments and the apparatus were adequate. There was nothing characteristic in two cats in which the condylar screw was inserted incorrectly on the left side—one was a 2-year-old DSH male weighing 4 kg, while the another one was a 12-year-old DLH female weighing 3 kg. Neither of them had a fracture of the humeral shaft.

## 4. Discussion

The purpose of this study was to investigate whether a medially inserted custom-made anatomically pre-shaped interlocking plate could be a useful implant for the reduction and stabilisation of Y-T humeral fractures in cats. Preparing this plate by applying computer-aided design to 3D printed bone models of domestic cats facilitated a plate morphology and screw orientation. The results show that even with comminution of the epicondylar region, anatomical reduction of intraarticular condylar fracture was easy and effective with such a plate. Most bone plates used in small animal trauma surgery have simple geometric shapes. Their use is time-consuming as they must be contoured intraoperatively to fit the patient’s anatomy. In addition, fixation screws must be carefully monitored to achieve primary stability while avoiding critical anatomy. The feline humeral condyle has a complex anatomy and some designs to create a more appropriate implant for the treatment of Y-T humeral fractures would be impossible to produce using traditional manufacturing processes. To investigate a solution to these practical challenges, this study quantified the accuracy associated with the design, manufacture and implantation of a 3D printed custom-made bone plate. Bone implants require a strong and biocompatible material, such as titanium alloy. Such material is difficult to use in traditional manufacturing due to its high melting point and other factors that prevent easy integration with traditional manufacturing techniques [21]. The laser beam sintering technique can produce very high-resolution details even when the materials do not alloy well or melt at the right temperature. Our study was conducted on forelimbs collected from adult domestic cats weighing between 3.0 and 4.5 kg. There was very little variability in humerus length and isthmus diameter between individuals in this group (as indicated by very low CV values), which means that the feline humerus appears to be a very good target for the development of anatomically pre-contoured locking bone plates. Unlike cats, dogs have a greater variability in the morphology of the skeleton, making it difficult to produce such implants for this species. We believe that anatomically pre-contoured implants for dogs can be developed only for specific breeds. To our knowledge, only Burton and colleagues [22] have successfully developed anatomically pre-contoured plates for the treatment of humeral condyle fractures in spaniels. In our study, we chose interlocking plates because the limited bone available in the region of the humeral condyle is the most limiting factor in fixation and restricts the options available for repair. In clinical cases, we also use interlocking plates to stabilise Y-T fractures with epicondylar comminution. Bilateral fixation with plates was considered to provide the greatest stability given the nature of the fractures. The parallel medial–lateral plating technique follows an arch-keystone concept like the principles of architecture in which two columns are anchored at their base and linked together at the top to provides stable fixation [23]. Locking internal fixation, which eliminates friction between the plate and bone, may be advantageous in the repair of humeral condylar fractures. Perfect plate contouring is not a prerequisite for accurate reduction of fracture fragments and fracture stability after fixation with an interlocking plate system. Similarly, the need for monocortical screws in each epicondylar ridge to avoid problems with screw tip impingement on the olecranon is also more appropriate for locking screw fixation, where the risk of monocortical screw loosening is reduced [24,25]. This is an important advantage of using interlocking plates, as loosening of monocortical cortical screws was the main cause of early catastrophic fixation failure in one of the five cats treated by Macias et al. [2]. A combined medial and lateral approach is mandatory for double plating of Y-T humeral fractures. Intraoperatively, care must be taken to avoid damaging the neurovascular structures. On the lateral side, there are branches of the radial nerve, while on the medial side are the brachial artery, brachial vein and the ulnar and median nerves. The supracondylar foramen is an important structure in the distal feline humerus as the brachial artery, brachial vein and median nerve pass through it [19]. These structures can be released from the supracondylar foramen if necessary for implant positioning. In all limbs prior to medial plating, the neurovascular structures were released from the epicondylar foramen after removing its medial border with rongeurs. This allowed the plate to be placed without damaging the vital structures. As our study was performed on cadavers, we were unable to assess the effect of neurovascular release on the postoperative period, but to the best of our knowledge, the negative effects of releasing neurovascular structures from the supracondylar foramen in cats have not been reported.

For most surgeons, the first step in reduction and fixation of the Y-T fracture depends on the configuration of the supracondylar fractures. In the case of comminution, the principle is to anatomically reduce and fix the condylar fragments, which is essential to minimise the risk of premature elbow arthritis [2]. This step effectively converts the T-condylar fracture into a supracondylar humeral fracture. The second step is the reduction and fixation of the supracondylar fracture line, ideally with a fixation that controls both the medial and lateral columns and allows early motion. In patients with reducible epicondylar fractures, it is possible to convert the bicondylar fracture to a monocondylar fracture [24,25]. We follow a similar treatment model for these fractures in our daily practice and believe that once a bicondylar fracture has been converted to a monocondylar fracture, the rest of the surgical procedure is easier to perform. This study showed that the custom-made anatomical pre-contoured plate acted as a template and allowed accurate fixation of the medial part of the condyle to the humeral diaphysis, even with comminution of the supracondylar region. After conversion of the bicondylar fracture to a monocondylar fracture, the lateral part of the humeral condyle and the comminuted fracture of the lateral epicondyle were successfully fixed.

Y-T fractures require precise anatomical reduction. The important intraoperative step is strong fixation of the intraarticular condylar fracture with a screw. The strategy to reduce the risk of screw failure (breaking or loosening) in the postoperative period is to use the screw with the largest thread core diameter that can be safely placed across the humeral condyle [22,26]. As a guide, a screw with a thread diameter of 30% to 50% of the diameter of the isthmus (narrowest part) of the humeral condyle should be used. In our study, the diameter of the isthmus of the humeral condyle ranged from 5.2 to 5.5 mm. Therefore, screws with a thread diameter of 2.0–2.7 mm fall within the range of 30–50% of the diameter of the narrowest part of the humeral condyle in operated limbs. The bending strength of a rod (screw’s core) is typically related to its moment of inertia, a property that depends on the geometry of the rod. For cylindrical rods, the moment of inertia is proportional to the fourth power of the radius (or diameter). To calculate the bending strength of a given diameter rod, the following formula for the moment of inertia of a circular section should be used: I = (π/64) × D^4^, where I is the moment of inertia and D is the diameter of the rod. Given this relationship, if diameter of the rod is increased, the increase in bending strength is exponential. Specifically, if diameter is doubled, the bending strength increases 16-fold (2^4^ times) [27]. These principles apply to all implants, e.g., nails, wires and screws. The evaluation of bone screws’ mechanical strength and bending strength can be simplified by taking the thread core diameter of the screw as the reference geometry. The following comparisons are based on moment of inertia calculations assuming material and other factors remain constant: Ø 2 mm screw—Ø 1.4 mm core, Ø 2.4 mm screw—Ø 1.7 mm core, and Ø 2.7 mm screw—Ø 1.9 mm core. The comparison of bending strengths for rods with diameters of 1.4 mm, 1.7 mm and 1.9 mm relative to the 1.4 mm diameter rod is as follows:

- A rod with a diameter of 1.4 mm has a baseline bending strength (considered as 1.0 for comparison);
- A rod with a diameter of 1.7 mm is approximately 2.17 times stronger in bending strength than a rod with a diameter of 1.4 mm;
- A rod with a diameter of 1.9 mm is approximately 3.39 times stronger in bending strength than a rod with a diameter of 1.4 mm.

Our prototype bone plate offered the option of using 2.0 mm, 2.4 mm and 2.7 mm threaded diameter screws. A 2.7 mm screw with a 1.9 mm core diameter was used to stabilise the humeral condyle as it had the highest mechanical strength compared to the 2.0 mm and 2.4 mm screws. Macias et al. [2] also used 2.7 mm transcondylar screws. In one case, such a transcondylar screw was replaced with a 3.5 mm screw as a salvage procedure due to stripping of the 2.7 mm screw. The authors of this study did not provide the diameter of the isthmus of the humeral condyle, so it is not known if this was a suitable implant for this cat.

Several studies have quantified and compared the biomechanical properties associated with lag screws and positional screws. A biomechanical study using simulated semicylindrical porcine rib fracture models demonstrated that preloaded positional screws (the compression force on the fragment is generated by a pair of bone-holding forceps) yield greater interfragmentary compression compared with lag screws [28]. Another biomechanical study using simulated intraarticular vertical femoral fracture models established that lag screws generate greater interfragmentary compressive force than positional screws, a contradiction of the previous result [29]. Differences in experimental equipment and methodology may account for the discrepancies in results. A biomechanical study in which oblique osteotomies were created in composite cortical bone models found that lag screws significantly increase interfragmentary compression generated by reduction forceps, whereas positional screws not only fail to maintain but significantly decrease the interfragmentary compression [30]. In contrast, Fuchter et al. [31] found that both cortical lag and cortical positional screws generated and maintained interfragmentary compression in a mature ovine humeral condylar fracture model. According to the authors, the increased interfragmentary compression and area of compression seen with the use of lag screws may make them the preferred option in mature bone, but this would require confirmation from a clinical trial. In small animal surgery, still no consensus on which implant is better for the fixation of humeral condylar fractures has been settled. Serrano Crehuet et al. [32] demonstrated that in canine cadaveric humeri mechanical properties were improved when the transcondylar osteotomy was stabilised with a 4.5 mm positional screw rather than a lag screw. In our study, the condyle was stabilised with pointed reduction forceps. The preloaded positional locking screw was then inserted and we achieved very good results in apposition and alignment of the humeral condyle. We believe that the differences described above between positional and lag screws may be due to the hardness and cohesion of the bones on which the tests were performed. A lag screw exerts very high pressure on the fragments when pressed into fragments with high mechanical strength. The insertion of a lag screw requires the drilling of a gliding hole. An under-drilled gliding hole will not compress the bone fragments, while an over-drilled hole will weaken the fixation. Given the limited bone mass available at the humeral condyle in cats, drilling the gliding hole requires a high degree of precision and may result in damage to this structure. The use of an anatomically pre-contoured plate reduces the need for wide exposure of the medial surface of the humeral condyle. The choice of locking screw technology meant that the plate did not have to be placed directly on the bone surface and the origin of the pronator teres could be preserved. Moreover, the use of a locking plate precludes the use of a locking screw as a lag screw. Based on our clinical experience (unpublished data), the use of preloaded positional screws to treat humeral condyle fractures in cats has good long-term clinical results.

Fracture of the humeral shaft is an additional complication in the treatment of Y-T humeral fractures. In reducible fractures, it is usually possible to reconstruct the shaft with cerclage wires. Cerclage wiring is not a suitable method as the sole method of stabilising long bone fractures, and support of the fracture with additional instrumentation is always mandatory. In our study, bicondylar humeral fractures were associated with a fracture of the humeral shaft in 7 of 30 limbs. After reconstruction of the humeral diaphysis with cerclage wiring as a support, a long lateral locking plate was used. In clinical cases of such complex fractures, the second lateral plate or external fixator may be added for additional stabilisation [25].

In only two of our cases (left limb of cat no. 4 and 11) was the screw incorrectly inserted into the humeral condyle. As the length of the humeral bones and the diameter of the isthmus of the humeral condyle did not differ from the other 28 operated bones, it can be assumed that the incorrect insertion of the implant was due to the incorrect application of the plate. In both cases, the screw was directed too caudally and did not reach the lateral part of the humeral condyle. Simpson and Meeson [3] defined the safe corridor for transcondylar screw placement in domestic cats and found that the tolerance angles were significantly lower when drilling in a medial to a lateral direction. Their study included an analysis of 20 CT scans of the elbows of 6 DSH, 1 DLH, 2 Maine Coons, 1 British Shorthair, 1 Exotic Shorthair and 1 Charteux cat. In this group of animals, the diameter of the humeral condyle isthmus ranged from 5.1 to 6.9 mm, which was similar to the range of humeral condyle isthmus diameters calculated in our study. As the medial surface of the humeral condyle and the medial epicondyle are covered when the plate is placed, the surgeon loses the anatomical landmarks, so it is important to place the plate correctly before starting osteosynthesis. Bongers et al. [33] found that the use of the 3D printed patient-specific drill guides could be challenging as a new surgical procedure, even for experienced surgeons familiar with anatomical landmarks. Our plate acts as a drill guide as well as a guide for bone implants (screws and wires). Although this reduces variation, it also limits tactile feedback when drilling implants, and an error in placement of the plate can affect the implants’ positions. As correct positioning of the pre-shaped bone plate is crucial, the Kirschner wire should be placed directly into the medial epicondyle, the 2 mm locking screw in the supracondylar region and the 2.7 mm locking positional screw in the center of the humeral condyle.

Our study has some limitations. The main limitation is that this study was conducted on isolated limbs with intact humeri. Fractures were created using a bone chisel and mallet. These included a Y-T fracture and epicondylar comminuted fractures. In humans, the mechanism of Y-T humeral fractures usually involves falling onto an outstretched arm with the elbow in slight flexion or directly onto the elbow. Both mechanisms involve splitting of the humeral condyles by a wedge action of the semilunar notch of the ulna against the trochlea of the humerus [14,15,34]. A similar mechanism of fracture would be expected in cats, so we used a bone chisel and mallet to split the humeral condyle in isolated limbs. We felt that the use of these tools mimicked the direction of action of the ulna splitting the humeral condyle in a real accident. As a result, we obtained fractures with a configuration similar to naturally occurring fractures in cats, described by [2,6,7]. The absence of post-traumatic changes associated with the fracture, such as swelling, haematoma and the absence of skeletal muscle contracture, may also be considered limitations. Therefore, clinical studies on the use of our pre-contoured plate are needed.

The final limitation is that there was very little variability in humerus length and isthmus diameter between individuals in our study group. This is likely due to the fact that 14/15 cats were DSH or DLH. Including a wider range of breeds would undoubtedly increase the diversity of anatomical circumstances in which the pre-contoured interlocking plate could be tested. On the other hand, DSH and DLH are the most widespread cat breeds in Poland, which makes our study group representative of the majority of clinical cases we may encounter in our daily practice.

## 5. Conclusions

Nowadays, additive manufacturing offers a wide range of possibilities for the creation of new surgical implants in human and veterinary medicine. The technology of metal powder sintering and CNC milling makes it possible to create a functional bone implant for the treatment of complex fractures. The custom-made pre-contoured bone plate appears to be a valuable tool in the reduction and stabilisation of bicondylar humeral fractures in adult domestic cats weighing 3.0 to 4.5 kg.

## Data Availability

The data presented in this study are available in the article.
